# Supplementary material for: Patterns of Intron Gain and Loss in Fungi
Source: PLoS Biol. 2004 Nov 30;2(12):e422. doi: 10.1371/journal.pbio.0020422 (PMC532390; doi:10.1371/journal.pbio.0020422)
Supplement: Table S1 — Also available at http://genes.mit.edu/NielsenEtAl/. (4.3 MB ZIP). [file pbio.0020422.st001.zip › NielsenEtAl/html/7.html]

AN0999.1.NCU08008.1.MG01722.1.FG01923.1


```
 CLUSTAL W (1.82) Multiple Sequence Alignments - Introns Inserted


Sequence 1: MG01722.1	533 aa
Sequence 2: FG01923.1	529 aa
Sequence 3: NCU08008.1	522 aa
Sequence 4: AN0999.1	529 aa
Alignment Length: 553 aa
Number Identitical Residues: 171 aa
Alignment Score (without introns) 9932


MG01722.1 	MATN-NMHNLTTLIKR2LEAATLRLEDIASSTID------LPQAVPSLSTTQPTPSATPA
NCU08008.1	MATN-NMHNLVTLIKR2LEAATLRLEDMASSTIP---------PIGSQAVIEPASSAPSA
FG01923.1 	MAVNSQMHNLTTLIKR2LEAATSRLEDIASATDPPADANVLNQAIPSP-LNPSSAAPPPA
AN0999.1  	MAATSHMHNLTTLIKR2LEAATSRLEDMAMSLDEH--------DAQKKTADNSAALEPQA
          	**..::****.***** ***** ****:* :               . :   .:.  . *

MG01722.1 	PGASAPTSTPPAAQDPLPESIEDFDLFITESVNNYANLSGKIGGPVAEQ0AKLLSKGFQL
NCU08008.1	PASTAVSTPAPAAAVPIPESVEKFDDFLNQTVQKYVKTSNLIGGLVAEQ0AASVLKGFAE
FG01923.1 	PTSNAAKPEPEAEAEPLPEAIEEFDAFLNTSVENYVKLSHQIGGLVAEQ0ASLVKTGFQE
AN0999.1  	SERPTPTAPAAPAAAPLPPQIEDFDTLISKDVGNFVQLGQKIGGLVAEQ0SNAVLEAFKA
          	.   : .. . .   *:*  :*.** ::.  * ::.: .  *** **** :  :  .*  

MG01722.1 	QRQFLLMTTKAKKPDMNGSDQSLWQDLLKPMSEVIMSVQGIKDANRGSEFFNHLSTTAEA
NCU08008.1	TRKFLLAASQSKKLDMNSVD---FQEAIKPISEAIQATQEIKDKNRGSPFFNHLGMVADG
FG01923.1 	QRKFLLISTKAKKPDLSGAGMPVFQDLIKPINEALMAVTELKDSNRPSPLITQLSTVSEG
AN0999.1  	ERTYLYVSTKAKKPEIQPPE------LMTGLHTASDAINNLRESNRASPLIDHLSAVAEG
          	 * :*  ::::** ::.          :. :  .  :   ::: ** * :: :*. .::.

MG01722.1 	VMVLAWVTVEPKPFKHVDVCMDSAQLFGNKVRMQYKEK2DPQHVQWVASLQQMCKDLAEY
NCU08008.1	IMLLAWITVQSRPDKLIDESLSSAEYLGNRVMQANKNT~DPQHTEWVKTFYQIFKDLSEY
FG01923.1 	IMVLAWVTVDARPFKHVDECLGAAQFFGNRVLKEYKEK2DPKQIEWVQSFYQVFRDLSDY
AN0999.1  	IVALGWF-FDPKPADFVSEMLGGIQYYGNKVLKEYKEK2DRTHYDYIQAYYQIFKSLSSY
          	:: *.*. .:.:* . :.  :.. :  **:*    *:. *  : ::: :  *: :.*:.*

MG01722.1 	IKQHFSNGLSWNPKG-----EPVQQVMKSMQSAPSGSPAPPPAPPAPS---AGGPPPPPP
NCU08008.1	AKDYYNNGIVWNPNG-----KPYKEVVQCLESGS--APSAPAASPAP----SGGAPPPPP
FG01923.1 	VKQYFSTGIPWNPQG-----QSVQEVLQSLSGAP-----APSAAPAPS---AGG-APPPP
AN0999.1  	LRKHYPKGLTWNDKDGIDAQEALRQVKNSSDSAAGGAGTPPPPPPVPTLNIPGGGAPPPP
          	 :.:: .*: ** :.. .:.:. ::* :. ......: :.*...*.*: . .** .****

MG01722.1 	PPPGPAPALKIKEQGAASTPAVTGINAVFSELNKGEAVTKGLRKVDKSEMTHKNPSLRAG
NCU08008.1	PPPGPPPVLKINEQKAEPAQP-GGFSSVMSELNKGEAVTKGLKKVDKSQMTHKNPSLRAG
FG01923.1 	PPPGPPPVLDIKTQEAPAAAPSGGLGAVFSELNKGDAVTKGLRKVDKSEMTHKNPSLRSG
AN0999.1  	PPPPPPPMISEQQ----SSGASSDMSAVFAQLNQGDAVTQGLRKVDKSQMTHKNPSLRAS
          	*** *.* :. :     .: .  .:.:*:::**:*:***:**:*****:*********:.

MG01722.1 	STVPEREGSVSSRGKSPAPG--KKPKPESMRVKKPPKKELDGNKWYI0ENFEQQPEPIEI
NCU08008.1	STVPD---GPAARSKSPAPPGTKPPKPESMRAKKPAKKALEGNKWII0ENYDAAAGNVEI
FG01923.1 	STV-----STSQRGKSPAPG--KKPKPESMRIKKPSKKELEGNKWTI0ENFEKEAEPIEI
AN0999.1  	STVPGSP-SSSSRSKSPAPS--KKPKPESMRSRKPPRKDLEGTKWFI~ENFDNPGDIVEI
          	***.    . : *.*****   * ******* :**.:* *:*.** * **::     :**

MG01722.1 	EANMSHSILISKCSGTTIRIHGKANAVTIENTSRVSLIIDSLVSTVDVVKSQNFGLQVMG
NCU08008.1	EASISQSILISRCNEVVIVVKGKANAVNIDNCDKVSLIVDTLVSTVDVVRSKNFQLQVNE
FG01923.1 	EASLTQSVLISRCNNTTVIVRGKANQVTVENSTRLSLIVDTLVSTVDVVKAQNFALQVMG
AN0999.1  	PAKQNQSILISRCNKTIIKVSSKANAIAIDNCHGLSIIVDSLVSSLDVVKCSKFALQIDG
          	 *. .:*:***:*. . : : .*** : ::*   :*:*:*:***::***:..:* **:  

MG01722.1 	TLPTVLMDQIDSAQVFFSRESASTRIFTSKSSNINVNVIG---ADDDYKELPLPSQMCHY
NCU08008.1	TIPTVLLDQVEQATIWLSKESISTRVFQSKTDNVNIMVVD---ENDDSKELPLPSQICSY
FG01923.1 	TIPTVMLDQIDSAQIYFSKESIGTKVFTSKSAGINLNVISG--EDEDYKEVPLPSQICSY
AN0999.1  	VAPTLLMDQVDGATVYLSSQSLGTELFSSKCTAVNVMLPPKEGTDEDTKECPVPEQIKTS
          	. **:::**:: * :::* :* .*.:* **   :*: :   .. ::* ** *:*.*:   

MG01722.1 	YDEAKGELVNEIVEHAG
NCU08008.1	YDPEKKELISEIVSHCG
FG01923.1 	YDEAKGDLVNEIVAHAG
AN0999.1  	VKD--GVLVSEIVEHAG
          	 .     *:.*** *.*
```
